# OUTPOST: A comprehensive analysis software for whole‐metagenome shotgun sequencing incorporating group stratification

**DOI:** 10.1002/imo2.29

**Published:** 2024-09-18

**Authors:** Yihang Zhou, Jihong Zheng, Wenqi Song, Xinyi Yan, Li Du, Zhonglin Ma, Yanbin Fu, Zhaohui Ouyang, Yuchen Xiao, Zhuoqun Liu, Feng Tian, Jason Wing Hon Wong, Jen Hao David Shih, Shikang Liang, Honglei Tian, Liu Liu, Ke Wei, Chao Zhang, Jiangtao Li, Xiaozhu Wang

**Affiliations:** ^1^ Fundamental Research Center, Shanghai Yangzhi Rehabilitation Hospital (Shanghai Sunshine Rehabilitation Center), School of Life Sciences and Technology Tongji University Shanghai China; ^2^ State Key Laboratory of Marine Geology, School of Ocean and Earth Science Tongji University Shanghai China; ^3^ Department of Biliary‐Pancreatic Surgery, Shanghai Cancer Institute Renji Hospital Affiliated to Shanghai Jiao Tong University School of Medicine Shanghai China; ^4^ State Key Laboratory of Cardiology and Medical Innovation Center, Shanghai East Hospital, Shanghai Key Laboratory of Signaling and Disease Research, Frontier Science Center for Stem Cell Research, School of Life Sciences and Technology Tongji University Shanghai China; ^5^ Hebei Key Laboratory of Medical Data Science, Institute of Biomedical Informatics, School of Medicine Hebei University of Engineering Handan China; ^6^ School of Biomedical Sciences, Li Ka Shing Faculty of Medicine The University of Hong Kong Pokfulam, Hong Kong, SAR China; ^7^ Division of Life Science The Hong Kong University of Science and Technology Clear Water Bay, Hong Kong, SAR China; ^8^ Shanghai Yuhui Pharmaceutical Technology (Group) Co., Ltd Shanghai China; ^9^ Shanghai Institute of Precision Medicine, Shanghai Ninth People's Hospital Shanghai Jiao Tong University School of Medicine Shanghai China

## Abstract

The whole metagenOme shotgun seqUencing sTream Pipeline that is cOmprehensive and uSeful for mulTi groups experiments (OUTPOST) is a comprehensive analysis software for whole‐metagenome shotgun sequencing incorporating group stratification, which encompasses 14 modules and boasts over 50 functions, distinguishing itself for its comprehensiveness when compared with 17 existing whole‐metagenome shotgun sequencing (WMGS) tools. OUTPOST introduces innovative methods for multi‐group experimental designs and meta‐analysis‐based biomarker identification.

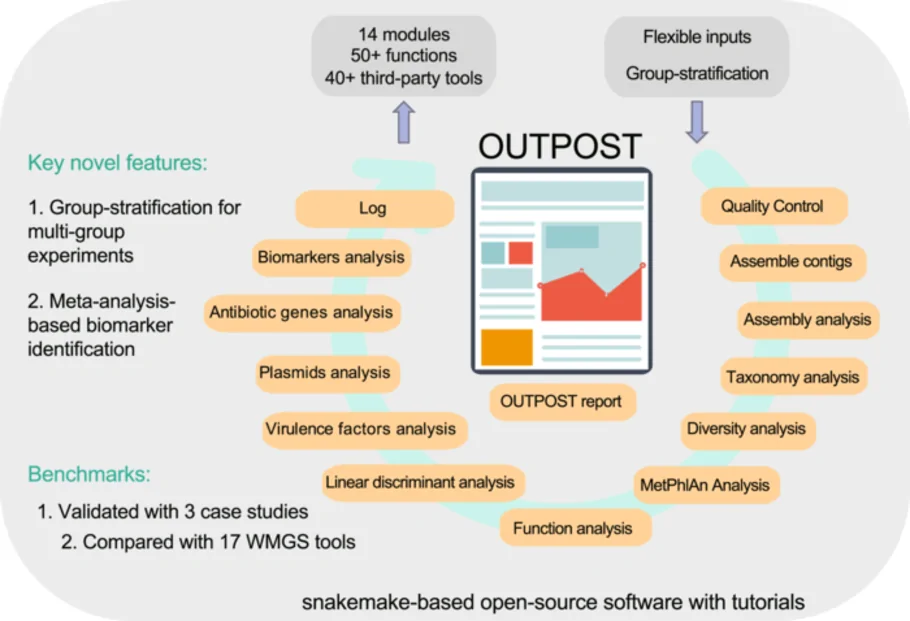

## ETHICS STATEMENT

No animals or humans were involved in this study.

To the Editors,

The field of microbiome research is expanding rapidly. Studying the role of microbiome in human health and disease can provide insights into precision medicine and therapeutic applications [1]. While rRNA sequencing lays the foundational work for understanding the microbiome [2], whole‐metagenome shotgun sequencing (WMGS) represents a pivotal advancement in microbiome research, offering an exhaustive and high‐resolution analysis of the microbiome [3, 4, 5]. This technology allows researchers to explore diverse aspects such as function [6, 7], taxonomy [8], antibiotics response [9], environmental quality [10], host interaction [11] and disease prediction [12]. However, WMGS data analysis is labor‐intensive and computationally demanding. It involves intricate upstream processes, including quality control of reads and assemblies, gene prediction, assembly alignment, and binning. Downstream analysis is also complex, encompassing taxonomy annotation and analysis, function annotation and analysis, diversity and phylogenetic analysis, statistical assessments, biomarker identification, antibiotics genes analysis, specialized investigations into antibiotic genes, virulence factors, and plasmids [13, 14]. This process includes various visualizations and additional specialized analyses. As the field of metagenomics evolves, experimental designs become more complex, with studies often featuring multiple distinct groups instead of just treatment and control groups. Analyzing data from such experiments requires intergroup comparisons at every analytical step, significantly complicating the analysis process.

Therefore, two primary issues exist in WMGS data analysis: inadequate comprehensiveness of current analysis pipelines and limited support for practical needs in experiments with multiple groups. To address the two existing challenges in WMGS data analysis, we introduced the whole metagenOme shotgun seqUencing sTream Pipeline that is cOmprehensive and uSeful for mulTi groups experiments (OUTPOST), a software designed to provide comprehensive analysis with deep adaptation for group stratification. OUTPOST comprises 14 modules, including 12 dedicated to analysis along with log and report modules. It enables users to assign multiple groups to each sample and automatically conducts intergroup comparisons across most analyses, featuring flexible input options and diverse outputs. To validate OUTPOST's effectiveness and robustness, we applied it to reanalyze a cat gut microbiome data set and successfully replicated most findings of the original paper. Extending its application, OUTPOST was used to analyze mammalian gut microbiomes, revealing significant differences between the microbiota of omnivores and herbivores. Furthermore, in a therapeutic context, this software was utilized to identify numerous biomarkers and functional virulence factors. The resulting mammalian and human microbiome assemblies have been made available for further research.

## 1 RESULTS

### 1.1 OUTPOST outstands for comprehensiveness

**Figure 1:**
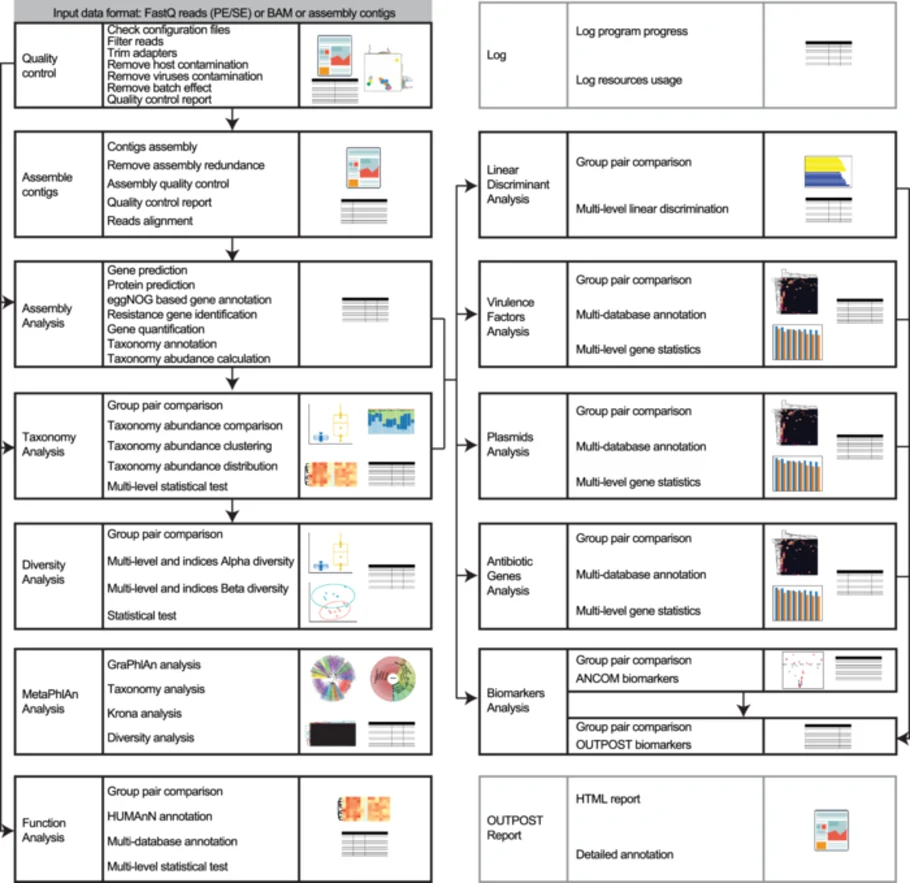
Sketch of cOmprehensive and uSeful for mulTi groups experiments (OUTPOST) workflow. OUTPOST is composed of 14 modules. For each block, the left cell contains the module name, the middle cell includes the function summary, and the right cell illustrates the output format. The lines and arrows indicate the data flow.

OUTPOST comprises 12 analysis modules, encompassing over 50 functions, providing more than 20 types of visualizations and numerous organized tables (Figure 1). Through meticulous engineering and design, it integrates and utilizes over 40 third‐party tools while offering many unique features (Tables [Link], [Link]). By comparing with 17 previous metagenomic analysis tools or pipelines, OUTPOST excelled in its comprehensiveness, covering a wide spectrum of analyses from quality control to biomarker analysis with a detailed HTML report (Table 1).

**Table 1:** cOmprehensive and uSeful for mulTi groups experiments (OUTPOST) comparing with other tools.

	A*	B*	C*	D*	E*	F*	G*	H*	I *	J*	K*	L*	M*	N*	O*	P*	Q*	R*
Reads as input	√	√		√	√	√	√	√	√	√	√	√	√	√	√	√	√	√
BAM files as input	√	√	√															
Contigs as input	√	√	√	√		√												
Group data handling	√																	
Multi‐group samples	√																	
Metadata verification	√			√					√		√							
Software requirements verification	√					√								√				
Reads quality control	√	√			√	√		√	√		√	√	√	√	√	√	√	√
Reads quality control report	√				√	√		√	√		√	√						
Host decontamination	√					√		√	√						√		√	
Virus decontamination	√								√		√							
Batch effect removal in taxonomy counts	√						√											
Contigs assembly	√	√	√	√	√	√		√			√	√	√	√	√	√	√	√
Assembly de‐redundance	√										√							
Assembly quality control	√			√	√	√		√		√	√	√				√	√	
Assembly quality control report	√			√	√	√		√			√	√					√	
Genes prediction from assembly	√				√			√			√	√	√	√	√	√	√	√
Proteins prediction from assembly	√				√								√		√		√	
EggNOG genes annotation from assembly	√				√								√				√	
Resistance genes identification from assembly	√	√			√									√				
Assembly genes quantification from assembly	√											√	√					
Read alignment to assembly	√	√				√		√	√	√	√		√		√	√		
Taxonomy annotation from assembly	√	√		√		√		√	√	√	√	√		√	√	√	√	√
Taxonomy abundance calculation	√								√		√					√	√	
Taxonomy analysis groups comparison	√																	
Taxonomy statistical test	√																	
Taxonomy clustering analysis	√								√				√					
Taxonomy distribution analysis	√																	
Taxonomy multi‐level statistical test	√																	
Diversity analysis comparison between groups	√																	
Multi‐level and indices Alpha diversity analysis	√					√			√	√	√							
Multi‐level and indices Beta diversity analysis	√					√			√	√	√							
Diversity statistical test	√																	
MetaPhlAn taxonomy analysis	√					√				√								
GraPhlAn analysis	√					√												
Phylogenetic analysis and visualization	√	√	√	√	√	√			√	√	√							
MetaPhlAn diversity analysis	√					√				√								
Function annotation from reads	√					√	√											
Function analysis groups comparison	√																	
Function item multi‐level statistical test	√																	
Linear discriminant analysis for taxa	√					√												
Linear discriminant analysis for functions	√																	
Antibiotics genes annotation from assembly	√	√		√	√								√	√				√
Antibiotics genes analysis groups comparison	√																	
Antibiotics genes multi‐level statistical test	√																	
Virulence factors annotation from assembly	√	√		√	√						√		√	√				√
Virulence factors analysis groups comparison	√																	
Virulence factors multi‐level statistical test	√																	
Plasmids annotation from assembly	√			√	√								√					√
Plasmids abundance analysis groups comparison	√																	
Plasmids multi‐level statistical test	√																	
Classical biomarker identification	√																	
Meta‐analysis based biomarker identification	√																	
Biomarkers analysis comparison between groups	√																	
Overall report generation	√	√	√	√	√				√		√			√	√			√
Workflow management	√		√			√					√						√	
Breakpoint resumption	√																√	
One‐line installation	√	√	√					√		√	√				√		√	√
Computational perturbation experiments	√																	
Multiple methods for robust analysis	√	√				√												
Open source software	√	√	√		√	√	√	√		√	√	√	√	√	√	√	√	√
Offering example data set and results	√	√	√			√	√			√	√	√		√		√		
Validation using existing data set	√	√	√	√		√	√	√	√	√	√	√	√	√	√	√	√	√

*This table uses letters to represent software name. A is OUTPOST, B is ASA3P, C is Anvi'o, D is BAP, E is BacSeq, F is bioBakery 3, G is HUMAnN3, H is IMP, I is iMAP, J is MetaPhlAn4, K is MetaPhage, L is MetaWRAP, M is MOCAT2, N is Nullarbor, O is PathoScope 2.0, P is SqueezeMeta, Q is Sunbeam, R is TORMES. OUTPOST: cOmprehensive and uSeful for mulTi groups experiments.

### 1.2 OUTPOST's novel features

#### 1.2.1 Designed for processing group stratification

Our approach to group stratification is similar to the capabilities found in Quantitative Insights Into Microbial Ecology 2 (QIIME2) [15] for the 16S sequencing, which processes metadata for analyzing multi‐group samples. OUTPOST's adaptation to group stratification is not an algorithm, but an engineering optimization, design, and effort applied to the software as a whole.

OUTPOST begins by assessing the appropriateness of group stratification, then automatically generates pairwise combinations for analysis. For each pair, it conducts intergroup comparisons across nearly all steps within modules such as Taxonomy analysis, Diversity analysis, Function analysis, Linear discriminant analysis, Virulence factors analysis, Plasmids analysis, Antibiotic genes analysis, and Biomarker analysis. These comparisons not only encompass statistical tests but also multi‐level calculations and visualizations of distributions and clustering, among others. For single‐group experiment, it automatically clones an identical group to ensure normal operation, a feature that does not compromise the analysis outcomes.

#### 1.2.2 Designed for computational perturbation experiments

**Figure 2:**
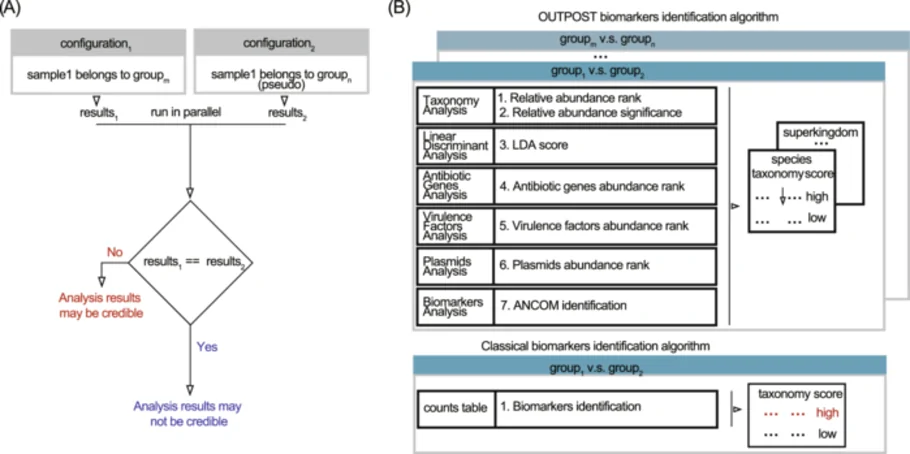
cOmprehensive and uSeful for mulTi groups experiments (OUTPOST) novel features. (A) Illustration for computational perturbation experiments using OUTPOST. (B) Illustration for meta‐analysis‐based OUTPOST's biomarker identification and classical biomarker identification algorithm.

In the realm of metagenomics analysis, most studies do not engage in perturbation studies, opting instead to merely present results based on current metadata. This contrasts with wet lab practices, where experiments such as knockouts are common to assess the robustness and credibility of their results, highlighting a community‐wide issue in metagenomics computational analysis. We propose that metagenomics analysis could also benefit from perturbation experiments, especially in designs with group information. As illustrated in Figure 2A, comparing results from actual groupings against those from pseudo‐groupings can gauge the reliability of the analysis. Similarity in outcomes suggests lower credibility, while differences indicate higher reliability.

OUTPOST is particularly well‐suited for conducting computational perturbation experiments. Unlike most metagenomic analysis tools, this software accounts for group stratification information, allowing for multiple groupings per sample. Additionally, it is designed for multi‐level intergroup comparisons, optimizing both the analysis workflow and result presentation. Conducting computational perturbation studies with our tool is straightforward, requiring only parallel runs in different working directories with varied metadata, without extending the analysis timeframe.

#### 1.2.3 Identifying biomarkers through meta‐analysis

Metagenomics studies frequently seek to identify biomarkers associated with specific phenotypes. Although numerous algorithms exist for biomarker identification, they often rely solely on a single source of information, typically a counts table. Our review indicates that most metagenomics studies do not depend solely on these algorithms for biomarker identification. Instead, they aggregate results from multiple analyses, statistically summarizing them to identify consistently significant microorganisms across various analyses as potential biomarkers. This approach fundamentally constitutes a meta‐analysis.

OUTPOST adopts a meta‐analytic approach to identify biomarkers (Figure 2B) by synthesizing results from seven types of analyses. It scores microorganisms based on their performance across each analysis type, ranking them accordingly. Microorganisms with high score are considered more likely to be biomarkers. Our software conducts this comparison across all taxonomy levels for every group pair.

#### 1.2.4 Robustness

OUTPOST exhibits robustness in two main aspects: its analysis workflow and its operational process. For analysis workflow robustness, this software ensures the reliability of its analysis results by diversifying its sources for mutual validation. For example, the taxonomy analysis results are derived independently from both our proprietary analysis pipeline and Metagenomic Phylogenetic Analysis (MetaPhlAn). Even within the same methods, OUTPOST diversifies data sources. For example, it calculates alpha diversity using both species‐ and genera‐level counts information. For operational process robustness, our software is engineered for reliability through thoughtful design. It employs Snakemake for workflow management, surpassing the limitations of simple script‐based calls. Capabilities for breakpoint resumption allow us to handle unforeseen interruptions. OUTPOST also automates resource allocation to enhance efficiency and continuously logs operational data. Additional design elements are integrated. These detailed engineering choices collectively fortify the operational robustness of our pipeline.

#### 1.2.5 Application and validation of OUTPOST in three cases

OUTPOST has been applied in three distinct metagenomics projects to demonstrate its utility and reliability in diverse contexts. In Case 1, we re‐analyzed an existing feline gut microbiome data set (Figure S1A‐C and S2‐4). Our reanalysis identified distinct microbial community distributions between normal and obese cats (Figure S1A). It also detected specific metabolic pathways and taxa associated with obesity (Figure S1B,C). The findings largely agree with the original study, while providing additional insights into the feline gut microbiome. In Case 2, OUTPOST was utilized for a mammalian gut microbiome data set featuring multi‐group stratification (Figure S1D‐G, S5, and Table S2). The analysis confirmed the tool's capability to manage datasets with complex experimental design (Figure S1D,E). The computational perturbation experiments validated the accuracy of OUTPOST's analysis (Figure S1F,G). Biomarkers distinguishing between omnivore and herbivore microbiomes were efficiently inferred, with further details provided in Table S3. In Case 3, we assessed a human gut microbiome data set, contrasting colorectal neoplasms with healthy samples, which carries greater medical significance than Case 1 (Figure S1H‐J, S6 and Table S4). OUTPOST identified potential bacterial markers and virulence factors associated with colorectal neoplasms, including *entD* in *Escherichia coli* (Figure S1H–J).

Detailed accounts of the methodologies (Figure S7) and results for these case studies are available in the supplementary material.

## 2 DISCUSSION

OUTPOST addresses the gaps in WMGS data analysis by enhancing the breadth of analytical functions and integrating group stratification information. Featuring 14 modules, it integrates over 40 third‐party tools and provides more than 50 functions. OUTPOST handles multiple groups per sample, enabling detailed automated comparisons across eight analysis modules. Designed with innovative features for computational perturbation experiments and biomarker identification via meta‐analysis, this software enhances the analysis's robustness and practical value. However, it is not designed to encompass all possible WMGS data analysis tasks, as this is nearly impossible given the continuous emergence of new methods. Nor does it aim to deeply develop any specific analysis function, as this falls outside OUTPOST's intended purpose. Originating from the practical requirements of WMGS research, our software strives to cover a broad spectrum of common analysis tasks, with a particular focus on accommodating multi‐group experimental designs and the identification of biomarkers.

We believe OUTPOST offers valuable assistance to users with varying levels of computational expertise, from novices to advanced researchers. For users unfamiliar with computing, OUTPOST serves as an installer for the WMGS analysis environment. By following tutorials on the software's website, users can deploy an environment with over 40 commonly used third‐party tools with a single line of code, saving considerable time and effort typically required for setup. For most users, OUTPOST acts as a gateway for WMGS data analysis, which allows for the execution of over 12 analysis modules, including unique meta‐analysis‐based biomarker identification, without concern for the complexities of multi‐group experimental designs. Additionally, there's no need to worry about interpreting voluminous results, as a detailed annotated report is generated by this pipeline. In the event of errors, the software's breakpoint resumption capability minimizes time loss. Advanced users will find our Snakemake‐based framework offers flexibility and extensibility, allowing them to extend OUTPOST's functionality by modifying the Snakefile. Moreover, OUTPOST tracks computational resource usage for each task, aiding in identifying time‐consuming bottlenecks. Additionally, its independence from third‐party tools allows advanced users to design their analysis workflows within the environment of our pipeline.

OUTPOST has its limitations. For instance, it does not include analyses related to binning, since we view binning as a distinct and complex analysis requiring extensive database installations, which tools like MetaWRAP [16] already address sufficiently, making it unnecessary for OUTPOST to incorporate binning. For further improvement, enhancing batch effect removal is a potential next step. Currently, our method for removing batch effects, inspired by HUMAnN, involves normalizing counts by grouped CPM (counts per million reads). Developing a more effective algorithm for WMGS batch effect removal could further standardize analysis within the field.

## 3 CONCLUSION

OUTPOST is a comprehensive analysis software for whole‐metagenome shotgun sequencing incorporating group stratification. With its 14 modules, including 12 dedicated to in‐depth analysis, it integrates over 50 functions and more than 40 third‐party tools, demonstrating superior comprehensiveness compared to 17 existing software solutions. Its novel approach to computational perturbation experiments, meta‐analysis for biomarker identification, and robust handling of multi‐group stratification underscores its utility and innovation. Validation through three diverse case studies showcases the ability of OUTPOST to replicate and extend findings from previous studies. The tool's operational robustness, facilitated by Snakemake and a modular script architecture, ensures efficient and reliable analysis.

## AUTHOR CONTRIBUTIONS

Xiaozhu Wang, Chao Zhang, Jiangtao Li and Ke Wei contributed to the conception and design of the study; Yihang Zhou, Jihong Zheng, and Wenqi Song built the tool, performed the data analysis and wrote the tutorial; Xinyi Yan, Li Du, Zhonglin Ma, Yanbin Fu, Zhaohui Ouyang, Yuchen Xiao, Zhuoqun Liu, Feng Tian and Liu Liu contributed to the tool improvement and data analysis; Jason Wing Hon Wong, Jen Hao David Shih, Shikang Liang, and Honglei Tian provided the computational methodology. Xiaozhu Wang supervised the study; Xiaozhu Wang and Yihang Zhou wrote the initial manuscript. All authors have read the final manuscript and approved it for publication.

## CONFLICT OF INTEREST STATEMENT

The authors declare no conflict of interest.

## Supporting information

The online version contains supplementary figures and tables available.

**Figure S1:** OUTPOST's validation.
**Figure S2:** OUTPOST comprehensively characterized the taxonomy atlas.
**Figure S3:** OUTPOST revealed significant different diversities and identified the potential features from the taxonomy and function analysis results.
**Figure S4:** OUTPOST reviewed the virulence factors, antibiotic genes, and plasmids crossing the taxonomy.
**Figure S5:** The multi‐group comparison indicated the significantly enriched abundance of Prevotella copri in omnivores, and the ablation experiment enhanced the reliability of the analyzed results.
**Figure S6:** The analysis of therapeutic microbiomes using OUTPOST.
**Figure S7:** The directed acyclic graph of OUTPOST Snakemake rules.

**Table S1:** List of software/database used in OUTPOST.
**Table S2:** The information of mammalian gut microbiome dataset.
**Table S3:** The OUTPOST's biomarker analysis results between omnivorous and herbivore gut microbiomes.
**Table S4:** The information of colorectal neoplasms gut microbiomes dataset.

## Data Availability

The data that support the findings of this study are openly available in OUTPOST at https://github.com/Y-H-Joe/OUTPOST. OUTPOST code is publicly available and can be found on GitHub alongside appropriate tutorials at https://github.com/Y-H-Joe/OUTPOST, and Zenodo (https://doi.org/10.5281/zenodo.11091729) with tutorials, examples and results. The WMGS data we used can be downloaded from NCBI (National Center for Biotechnology Information) [17] under the accession number in Table S2 (mammalian data set) and Table S4 (colorectal neoplasms data set). An example of an HTML report is available at https://github.com/Y-H-Joe/OUTPOST/blob/main/example_data/Data_S1_OUTPOST_report_example.7z. Supplementary information (methods, figures, tables, slides, videos, Chinese translated version, and updated materials) may be found in the online DOI or iMeta Science http://www.imeta.science/imetaomics/.

## References

[1] Lynch, Susan V. , and Oluf Pedersen . 2016. “The Human Intestinal Microbiome in Health and Disease.” New England Journal of Medicine 375: 2369–2379. 10.1056/NEJMra1600266 27974040

[2] Liu, Yong‐Xin , Yuan Qin , Tong Chen , Meiping Lu , Xubo Qian , Xiaoxuan Guo , and Yang Bai . 2021. “A Practical Guide to Amplicon and Metagenomic Analysis of Microbiome Data.” Protein Cell 12: 315–330. 10.1007/s13238-020-00724-8 PMC8106563 32394199

[3] Turnbaugh, Peter J. , Ruth E. Ley , Micah Hamady , Claire M. Fraser‐Liggett , Rob Knight , and Jeffrey I. Gordon . 2007. “The Human Microbiome Project.” Nature 449: 804–810. 10.1038/nature06244 PMC3709439 17943116

[4] Yatsunenko, Tanya , Federico E. Rey , Mark J. Manary , Indi Trehan , Maria Gloria Dominguez‐Bello , Monica Contreras , Magda Magris , et al. 2012. “Human Gut Microbiome Viewed Across Age and Geography.” Nature 486: 222–227. 10.1038/nature11053 PMC3376388 22699611

[5] Ma, Xiaolei , Emily Brinker , Emily C. Graff , Wenqi Cao , Amanda L. Gross , Aime K. Johnson , Chao Zhang , Douglas R. Martin , and Xu Wang . 2022. “Whole‐Genome Shotgun Metagenomic Sequencing Reveals Distinct Gut Microbiome Signatures of Obese Cats.” Microbiology Spectrum 10: e0083722. 10.1128/spectrum.00837-22 PMC9241680 35467389

[6] Wang, Wei‐Lin , Shao‐Yan Xu , Zhi‐Gang Ren , Liang Tao , Jian‐Wen Jiang , and Shu‐Sen Zheng . 2015. “Application of Metagenomics in the Human Gut Microbiome.” World Journal of Gastroenterology 21: 803–814. 10.3748/wjg.v21.i3.803 PMC4299332 25624713

[7] Yang, Jian , Peng Zheng , Yifan Li , Jing Wu , Xunmin Tan , Jingjing Zhou , Zuoli Sun , et al. 2020. “Landscapes of Bacterial and Metabolic Signatures and Their Interaction in Major Depressive Disorders.” Science Advances 6: eaba8555. 10.1126/sciadv.aba8555 PMC7710361 33268363

[8] Forster, Samuel C. , Nitin Kumar , Blessing O. Anonye , Alexandre Almeida , Elisa Viciani , Mark D. Stares , Matthew Dunn , et al. 2019. “A Human Gut Bacterial Genome and Culture Collection for Improved Metagenomic Analyses.” Nature Biotechnology 37: 186–192. 10.1038/s41587-018-0009-7 PMC6785715 30718869

[9] Guo, Jianhua , Jie Li , Hui Chen , Philip L. Bond , and Zhiguo Yuan . 2017. “Metagenomic Analysis Reveals Wastewater Treatment Plants as Hotspots of Antibiotic Resistance Genes and Mobile Genetic Elements.” Water Research 123: 468–478. 10.1016/j.watres.2017.07.002 28689130

[10] Breton‐Deval, Luz , Alejandro Sanchez‐Flores , Katy Juárez , and Rosario Vera‐Estrella . 2019. “Integrative Study of Microbial Community Dynamics and Water Quality Along the Apatlaco River.” Environmental Pollution 255: 113158. 10.1016/j.envpol.2019.113158 31521989

[11] Kishikawa, Toshihiro , Yuichi Maeda , Takuro Nii , Daisuke Motooka , Yuki Matsumoto , Masato Matsushita , Hidetoshi Matsuoka , et al. 2020. “Metagenome‐Wide Association Study of Gut Microbiome Revealed Novel Aetiology of Rheumatoid Arthritis in the Japanese Population.” Annals of the Rheumatic Diseases 79: 103–111. 10.1136/annrheumdis-2019-215743 PMC6937407 31699813

[12] Loomba, Rohit , Victor Seguritan , Weizhong Li , Tao Long , Niels Klitgord , Archana Bhatt , Parambir Singh Dulai , et al. 2017. “Gut Microbiome‐Based Metagenomic Signature for Non‐Invasive Detection of Advanced Fibrosis in Human Nonalcoholic Fatty Liver Disease.” Cell Metabolism 25: 1054–1062.e5. 10.1016/j.cmet.2017.04.001 PMC5502730 28467925

[13] Quince, Christopher , Alan W. Walker , Jared T. Simpson , Nicholas J. Loman , and Nicola Segata . 2017. “Shotgun Metagenomics, from Sampling to Analysis.” Nature Biotechnology 35: 833–844. 10.1038/nbt.3935 28898207

[14] Sedlar, Karel , Kristyna Kupkova , and Ivo Provaznik . 2017. “Bioinformatics Strategies for Taxonomy Independent Binning and Visualization of Sequences in Shotgun Metagenomics.” Computational and Structural Biotechnology Journal 15: 48–55. 10.1016/j.csbj.2016.11.005 PMC5148923 27980708

[15] Bolyen, Evan , Jai Ram Rideout , Matthew R. Dillon , Nicholas A. Bokulich , Christian C. Abnet , Gabriel A. Al‐Ghalith , Harriet Alexander , et al. 2019. “Reproducible, Interactive, Scalable and Extensible Microbiome Data Science Using QIIME 2.” Nature Biotechnology 37: 852–857. 10.1038/s41587-019-0209-9 PMC7015180 31341288

[16] Uritskiy, Gherman V. , Jocelyne DiRuggiero , and James Taylor . 2018. “MetaWRAP‐a Flexible Pipeline for Genome‐Resolved Metagenomic Data Analysis.” Microbiome 6: 158. 10.1186/s40168-018-0541-1 PMC6138922 30219103

[17] Sayers, Eric W. , Richa Agarwala , Evan E. Bolton , J. Rodney Brister , Kathi Canese , Karen Clark , Ryan Connor , et al. 2019. “Database Resources of The National Center for Biotechnology Information.” Nucleic Acids Research 47: D23–D28. 10.1093/nar/gky1069 PMC6323993 30395293

