# Supplementary material for: OUTPOST: A comprehensive analysis software for whole‐metagenome shotgun sequencing incorporating group stratification
Source: IMetaOmics. 2024 Sep 18;1(2):e29. doi: 10.1002/imo2.29 (PMC12806516; doi:10.1002/imo2.29)
Supplement: Supplementary file 1 — Figure S1: OUTPOST's validation. Figure S2: OUTPOST comprehensively characterized the taxonomy atlas. Figure S3: OUTPOST revealed significant different diversities and identified the potential features from the taxonomy and function analysis results. Figure S4: OUTPOST reviewed the virulence factors, antibiotic genes, and plasmids crossing the taxonomy. Figure S5: The multi‐group comparison indicated the significantly enriched abundance of Prevotella copri in omnivores, and the ablation experiment enhanced the reliability of the analyzed results. Figure S6: The analysis of therapeutic microbiomes using OUTPOST. Figure S7: The directed acyclic graph of OUTPOST Snakemake rules. [file IMO2-1-e29-s001.docx]

**Supporting information to** **OUTPOST: a comprehensive analysis software for whole-metagenome shotgun sequencing incorporating group stratification**

**Running title:** OUTPOST: Comprehensive WMGS analysis with group stratification

Yihang Zhou^1#^, Jihong Zheng^1#^, Wenqi Song^1#^, Xinyi Yan^1^, Li Du^1^, Zhonglin Ma^2^, Yanbin Fu^3^, Zhaohui Ouyang^4^, Yuchen Xiao^1^, Zhuoqun Liu^1^, Feng Tian^5^, Jason W.H. Wong^6^, David J.H. Shih^6^, Shikang Liang^6^, Honglei Tian^7^, Liu Liu^8^, Ke Wei^4*^, Chao Zhang^1*^, Jiangtao Li^2*^, Xiaozhu Wang^1, 9*^

^1^Fundamental Research Center, Shanghai Yangzhi Rehabilitation Hospital (Shanghai Sunshine Rehabilitation Center), School of Life Sciences and Technology, Tongji University, Shanghai 201619, China

^2^State Key Laboratory of Marine Geology, School of Ocean and Earth Science, Tongji University, Shanghai 200092, China

^3^Shanghai Cancer Institute, Department of Biliary-Pancreatic Surgery, Renji Hospital Affiliated to Shanghai Jiao Tong University School of Medicine, Shanghai 200127, China

^4^State Key Laboratory of Cardiology and Medical Innovation Center, Shanghai East Hospital, Shanghai Key Laboratory of Signaling and Disease Research, Frontier Science Center for Stem Cell Research, School of Life Sciences and Technology, Tongji University, Shanghai 200120, China

^5^Hebei Key Laboratory of Medical Data Science, Institute of Biomedical Informatics, School of Medicine, Hebei University of Engineering, Handan 056038, China

^6^School of Biomedical Sciences, Li Ka Shing Faculty of Medicine, The University of Hong Kong, Pokfulam, Hong Kong SAR 999077, China

^7^Division of Life Science, The Hong Kong University of Science and Technology, Clear Water Bay, Hong Kong SAR 999077, China

^8^Shanghai Yuhui Pharmaceutical Technology (Group) Co., Ltd., and Shanghai Ruishen Technology Development Co., Ltd, Shanghai 201203, China

^9^Shanghai Institute of Precision Medicine, Shanghai Ninth People's Hospital, Shanghai Jiao Tong University School of Medicine, Shanghai 200125, China

^#^These authors contributed equally: Yihang Zhou, Jihong Zheng, and Wenqi Song.

*Correspondence: [wangxiaozhu@sjtu.edu.cn](mailto:wangxiaozhu@sjtu.edu.cn) (Xiaozhu Wang), [jtli@tongji.edu.cn](mailto:jtli@tongji.edu.cn) (Jiangtao Li), [zhangchao@tongji.edu.cn](mailto:zhangchao@tongji.edu.cn) (Chao Zhang), and [kewei@tongji.edu.cn](mailto:kewei@tongji.edu.cn) (Ke Wei)

**Supplementary contents**

**Application and validation of OUTPOST in three cases**

In the realm of WMGS data analysis, existing tools often lack comprehensiveness or multi-group data handling capabilities. Some tools are well-developed for specific purposes but lack comprehensive analysis capabilities, whereas others offer extensive analytical functions but fall short in handling multi-group data. Over the years, many notable and powerful software have been developed, such as ASA3P [1], Anvi’o [2], BAP [3], BacSeq [4], IMP [5], iMAP [6], MetaPhage [7], MetaWRAP [8], MOCAT2 [9], Nullarbor [10], PathoScope 2.0 [11], SqueezeMeta [12], Sunbeam [13], and TORMES [14]. Among these, bioBakery3 [15] stands out as one of the most comprehensive integrations of taxonomic, functional, and strain-level profiling. It includes components like KneadData, ChocoPhlAn 3, PhyloPhlAn 3 [16], PanPhlAn 3, StrainPhlAn 3 [17], MetaPhlAn 3 [18], and HUMAnN 3 [19]. Despite the extensive suite of tools, these tools do not offer deep adaptation for multi-group experiments. Therefore, two primary issues exist in WMGS data analysis: inadequate comprehensiveness of current analysis pipelines and limited support for practical needs in experiments with multiple groups.

To address the two existing challenges in WMGS data analysis, we introduced OUTPOST (the whole metagenOme shotgun seqUencing sTream Pipeline that is cOmprehensive and uSeful for mulTi groups experiments) compared with 17 previous tools or pipelines [1-15, 18], and applied it on three cases (Figure S1 and Table S1).

**Case 1: The evaluation of OUTPOST using the published cat microbiome work**

The taxonomy analysis reproduced the bacterial distribution and statistical test in prior work [20]. This analysis generated bar plots displaying relative frequency at all taxonomy levels. At the phylum level, we observed that more than 95% of the microbes belonged to Firmicutes, Bacteroidetes, Actinobacteria, Proteobacteria, and Fusobacteria (Figure S2A). OUTPOST performed unsupervised clustering of the relative abundance of the top 20 families, which formed two distinct groups for the normal and obese cat microbiomes (Figure S2B). To screen potential biomarkers, OUTPOST statistically tested taxa crossing all levels. We observed a significant relative abundance decrease from normal to obese cat microbiomes in Firmicutes (phylum), Clostridia (class), Eubacteriales (order), Lachnospiraceae (family), and *Lactimicrobium massiliense* (species). Bifidobacterium (genus) significantly increased in obese cat microbiome (Figure S2C−H). According to our knowledge, researchers in previous studies had to manually select and calibrate target taxa, which is laborious and may miss important information. OUTPOST, on the contrary, automatically screened and produced 14,934 boxplots for all statistically different taxa crossing 8 levels (superkingdom, phylum, class, order, family, genus, species, and taxonomy ID), which advanced the former work [20].

OUTPOST's diversity analysis replicated the original conclusions while emphasizing potential fragility in conclusions drawn from alpha diversity differences. Using various methods, OUTPOST computed both alpha and beta diversity for all pairwise comparisons, revealing significant differences between normal and obese cat microbiomes at the species and genus levels. The Shannon index showed a decrease in microbiota diversity, but this significance vanished when calculated using the Simpson index (Figure S3A, B). A noteworthy difference was driven by the second principal component (Figure S3C, D), highlighting underlying factors unrelated to overall abundance or diversity. Considering that many studies rely on a single index, the five indexes processed by OUTPOST bolster conclusion robustness.

The function analysis characterized the metabolic pathway composition, unveiling a lack of distinct consistency between normal and obese cat microbiomes. OUTPOST's statistical analysis and visualization (Figure S3E, F) pointed to the prominence of protein synthesis in cat microbiome function, with subunit ribosomal proteins constituting 18 of the top 20 most abundant KEGG Orthology terms.

Linear discriminant analysis results from OUTPOST were in line with prior work in taxonomy analysis, reaffirming features of specific orders and genera (Figure S3G, H) and the specificity of Bifidobacterium. In terms of function, OUTPOST identified two of the featured enzymes among the top 3 abundant unequal enzymes (EC 3.2.1.23 and EC 6.3.5.5) (Figure S3I), while also providing supplementary insights into linear discriminant features from five independent function databases. These consistent findings corroborate previous research while adding further layers of understanding to the subject.

The analysis of virulence factors, antibiotic genes, and plasmids was absent in the former work [20], yet OUTPOST provided novel findings on the distribution and abundance of these factors in cat microbiomes. We counted the top 10 virulence factors and found clpV had the widest distribution among species. The species Clostridium sp. CAG:169, Collinsella tanakaei, Coriobacteriia bacterium and Thermophilibacter provencensis carried 3 out of the top 10 virulence factors, while the rest carried only one or two factors (Figure S4A). For antibiotic genes, lnuC and gene1 had the widest distribution among families. The family Bacteroidaceae carried the most antibiotic genes (Figure S4B). In general, the distribution of virulence factors and antibiotic genes was sparse and concentrated in cat microbiota. Strikingly, all top-10 virulence factors were found to have a higher abundance in the normal cat microbiome compared to the obese cat microbiome (Figure S4C). This biased distribution in the normal group was also observed in plasmids (Figure S4D).

**Case 2: The further exploitation of OUTPOST with the mammalian microbiome**

To demonstrate the reliability of utilizing OUTPOST for computational ablation experiments, we intentionally provided incorrect grouping information to observe whether the controlled analysis results remained valid under erroneous groupings. OUTPOST first characterized the microbial profile (Figure S5A-H, Table S2). In taxonomy analysis, we found the top 20 most abundant species formed distinct groups for omnivore and herbivore microbiomes (Figure S5I), while this difference disappeared in the ablation group (Figure S5J). The significance of distinct beta diversity between omnivore and herbivore microbiomes also vanished (Figure S5K, L). Under incorrect group information, the original conclusions no longer existed, validating their reliability. With OUTPOST, the entire computational ablation experiment required only a few additional minutes for preparing a new configuration table.

To demonstrate the value of OUTPOST in biomarkers inference, we integrated results from previous analytic modules and scored biomarkers across all taxonomic categories. This was performed in a comparison between omnivorous and herbivorous organisms. At the species level, Prevotella copri obtained the highest biomarker score of 7, which stands for ranking among the top 100 abundant species with significant relative abundance differences, high LDA score, identification by ANCOM, and abundant virulence factors genes, plasmids, and antibiotic genes. At other taxonomic levels, OUTPOST pinpointed biomarkers such as Arthrobacter, Flavonifractor, Oscillibacter, and Ruminococcus at the genus level, various families, orders, classes, and phyla as outlined (Table S3). The ANCOM results, a part of the scoring criteria, were also depicted in a volcano plot.

**Case 3: The test of OUTPOST in therapeutic microbiome**

To demonstrate the effectiveness of OUTPOST in analyzing therapeutic microbiomes, we analyzed a randomly collected dataset of gut microbiomes from patients with colorectal neoplasms and healthy individuals (Table S4). Colorectal neoplasm, a common cancer, is the third leading cause of cancer-related deaths worldwide [21]. A growing number of works uncovered the relationship between gut microbiomes and colorectal neoplasms [22-25]. Previous studies have identified potential bacterial markers for colorectal neoplasms microbiota, such as Escherichia coli [26-28], Prevotella intermedia [29-31], Peptostreptococcus anaerobius [32, 33], Parvimonas micra [34-36], Fusobacterium nucleatum [37-39], and Alistipes finegoldii [31, 40]. These were successfully identified by OUTPOST in patient gut microbiotas compared to healthy ones (Figure S6A-F).

Furthermore, OUTPOST detailed the top virulence factors and their corresponding species, with Escherichia coli displaying the broadest range of virulence factors (Figure S6G). Of note, OUTPOST pinpointed entD as the most abundant and sharply increased virulence factor in patients' microbiomes (Figure S6H). Interestingly, entD was also the dominant virulence factor in Escherichia coli, as well as plays a vital role in producing enterobactin, an iron-chelating bacterial siderophore known to inhibit cancer cell proliferation [41]. Recent studies have emphasized enterobactin's role in colorectal cancer development [42, 43]. Utilizing OUTPOST, we not only replicated existing bacterial biomarkers but also unveiled the potential impact of enterobactin—possibly driven by the virulence factor entD in Escherichia coli—on the progression of colorectal neoplasms.

**OUTPOST pipeline directed graph**

In Figure S7, we presented the processing workflow of OUTPOST, which integrates 14 modules encompassing over 50 functions and more than 40 third-party tools. This workflow facilitates a comprehensive approach to data analysis, ranging from quality control to biomarker identification. The figure demonstrates how OUTPOST's structured pipeline aids in the integration and analysis of complex datasets, offering detailed insights and supporting downstream applications through a comprehensive HTML report.

**Supplementary methods**

**Software implementation**

OUTPOST is a Snakemake [44] based pipeline developed using Python, R, Bash, and third-party tools. The pipeline consists of 14 modules, including 12 analytical modules, one log module, and one report module (Figure S7).

The installation process has been streamlined to a single-line command, demonstrating a commitment to ease of use and broad accessibility. Engineered for compatibility with GNU/Linux operating systems, OUTPOST can be effortlessly installed on individual personal computers or deployed on computational cluster nodes, circumventing the need for administrative privileges.

The execution requires specifying parameter values within a YAML-formatted file. The program embarks on a systematic verification process, initially assessing the compliance of the configuration table with predefined standards, followed by an evaluation of the parameter settings for correctness, before proceeding to execution. OUTPOST can operate in a multi-core parallel processing mode, allocating CPU resources for computational subtasks automatically. Furthermore, it generates comprehensive logs detailing the utilization of computational resources, alongside progress reports for ongoing tasks. Notably, this software features an advanced breakpoint resumption functionality, allowing it to autonomously detect and skip workflow components that have been previously completed, thus optimizing computational efficiency.

The input requirements encompass a dataset and a configuration table. This dataset can be sequenced reads (both paired-end and single-end formats are supported), BAM files (which are the aligned sequences against an assembly), assembly contigs, or a combination of these. The configuration table provides essential metadata for each sample, incorporating details such as group stratification, allowing for the association of each sample with multiple groups.

The output is organized into a dedicated directory structure, with each analytical module corresponding to a specific subdirectory. The formats of the output include vector plot PDFs for graphical data representation, supplementary tables, and a comprehensive report in HTML format. This structured approach facilitates the generation of a concise report, replete with detailed annotations for user reference.

OUTPOST incorporates group stratification information, where most of its analytic modules automatically perform analyses across all pairwise group comparisons. This inter-group analysis includes both statistical testing and comparative analysis of results between groups. Horizontally, OUTPOST's inter-group analysis encompasses all group pairs, while vertically, it covers the vast majority of analytical levels, integrating and synthesizing findings across these dimensions.

OUTPOST integrates over 40 third-party tools, maintaining their operational independence within its framework. This design philosophy ensures that users are not confined to predefined analytical pipelines. Instead, it affords the flexibility to customize or develop novel workflows within the OUTPOST environment, significantly reducing the complexities associated with the installation and configuration of a sophisticated computational infrastructure.

**Software architecture**

**Quality control**

OUTPOST checks the configuration table for sample validity and input file presence before conducting quality control (QC) on input reads. Users can decide whether to downsample reads to accelerate the whole process. It then uses fastp [45] for eliminating low-quality bases and adapters, generating a QC report, and further employs Trimmomatic [46] alongside OUTPOST's self-collected adapter library for further adapter removal. Our pipeline facilitates host contamination removal, allowing users to specify different hosts per sample and automatically index unindexed host genomes. It addresses viral contamination using its viral genome library. Post-cleaning, the software aligns reads to the assembly, calculates counts, removes batch effects given group stratification, and visualizes variance distribution.

The workflow adapts to user specifications, skipping host contamination removal if the host genome is unspecified and allowing configurable viral contamination and batch effect removal parameters. If the input data is non-read, OUTPOST will automatically bypass read cleaning steps.

**Assemble contigs**

OUTPOST assembles contigs using MEGAHIT [47] or metaSPAdes [48]. It then employs CD-HIT-EST [49] to remove redundant contigs. The assembly step is skipped if a user-provided assembly is present. Contigs undergo quality assessment and reporting through QUAST [50]. Finally, it aligns cleaned reads to the assembly, producing BAM files for subsequent analysis.

**Assembly analysis**

OUTPOST predicts genes and proteins on assemblies using MetaGeneMark and Prodigal, and generates GTF files automatically for users. It annotates genes using eggNOG-mapper based on the EggNOG [51] database and further annotates genes with RGI [52] based on the CARD [52] database. Gene quantification is performed using Salmon. For species annotation of all assembly contigs, it uses Kaiju [53] with the annotation level specified by the user.

OUTPOST also provides statistical tests for assembly contigs, which is helpful for binning. For each group pair, the *p*-value and q-value of all contigs are calculated based on counts, allowing users to manually choose contigs for further MAG (metagenome-assembled genome) assembly based on statistical results and counts. Our pipeline offers Bonferroni (equation (1)), Bonferroni-Holm (equation (2)), and Benjamini-Hochberg (equation (3)) corrections. OUTPOST keeps the significance level in these equations unchanged (0.05) and calculates the *p*-value via the inverse function of the *p*-value adjustment methods.

|  | $q_{g}=P_{g}\times m$ | (1) |
| --- | --- | --- |
|  | $q_{g}=P_{g}\times(m-rank_{g})$ | (2) |
|  | $q_{rank_{g}}=\left\{ \begin{aligned} P_{rank_{g}}\times\frac{m}{rank_{g}}, &q_{rank_{g}}\leq q_{rank_{g}+1} \\ q_{rank_{g}+1}, &q_{rank_{g}}>q_{rank_{g}+1} \end{aligned} \right.$ | (3) |

The *q_g_* is the q-value of contig *g*, *P_g_* is the *p*-value of contig *g*, *m* is the number of contigs and *rank_g_* is the rank of *P_g_* in an ascending sort. The statistical test is optional for users.

**Taxonomy analysis**

OUTPOST uses the idxstats function in SAMtools [54] to summarize the counts for every microbial contig crossing samples. It then employs Kaiju [53] to annotate the taxonomy of contigs at various levels, from superkingdom to species and NCBI taxonomy ID. Users can optionally remove the batch effects by CPM (counts per million reads) normalization given the batch information. For one batch, let *C* be the counts of contig *g* in sample s, *n* be the number of samples in this batch, and *m* be the number of contigs in the assembly:

|  | $C_{s,g}^{'}=\frac{C_{s,g}}{\sum_{s=1}^{n} \sum_{g=1}^{m} C_{s,g}}\times1,000,000$ | (4) |
| --- | --- | --- |

For further analysis and comparison, OUTPOST transfers the CPM value to relative abundance *A* for each taxonomic level:

|  | $A_{s,t}=\frac{C_{s,t}^{'}}{\sum_{t=1}^{l} C_{s,t}^{'}}$ | (5) |
| --- | --- | --- |

The *t* is the taxon and *l* is the number of taxa in sample s. As equation (5) indicates, OUTPOST excludes the contigs without taxonomy annotation in the calculation. The software selects statistical methods for testing group pairs based on the experimental design. For paired groups, it uses the two-tailed or one-tailed Wilcoxon signed-rank test. For unpaired groups, it applies the two-tailed or one-tailed Mann–Whitney U test.

We offer various visualization results in this tool based on the taxonomy abundance clustering, distribution, and multi-level statistical tests.

**Diversity analysis**

OUTPOST calculates the diversity based on taxonomy relative abundance at the species and genus levels using vegan [55] and custom scripts. The alpha diversity indices include the Simpson index, Shannon index, Pielou index, and their derivatives. The beta diversity indices include the Bray-Curtis index and the Jaccard index. It then visualizes the analysis results using box plots and principal coordinate analysis (PCoA) plots, with statistical tests.

**MetaPhlAn analysis**

OUTPOST employs MetaPhlAn4 [18] for taxonomy-related analysis directly from reads, enhancing the robustness of its analysis independently. It goes beyond merely invoking MetaPhlAn by updating its source code for improved functionality. Our software recalculates taxonomy abundance and subsequently computes new alpha and beta diversity indices. Furthermore, it analyzes and visualizes taxonomy and abundance from a phylogenetic perspective using GraPhlAn [56] and Krona [57].

**Function analysis**

OUTPOST uses HUMAnN3 [15] to annotate function tables from cleaned reads for each sample. The function tables are counts of features across five independent databases by default: MetaCyc [58], KEGG Orthology [59], Pfam [60], ENZYME [61], and EggNOG [51]. It normalizes the feature counts by CPM for each sample. To maintain consistency with taxonomy analysis, the CPM values are further converted to relative frequency. Multi-level statistical tests are conducted. Cluster heatmaps are used to visualize the statistical test results and relative frequency distribution.

**Linear discriminant analysis**

With the relative frequency tables from the taxonomy and function analysis, OUTPOST applies linear discriminant analysis across all taxonomy levels and function items. It calculates the LDA scores using modified scripts from LEfSe [62] and generates bar plots for features with scores exceeding the parametric threshold.

**Virulence factors analysis**

The virulence factors analysis, antibiotic genes analysis, and plasmids analysis link taxa with specific genes and profile the distribution of genes across samples. Innovatively, OUTPOST connects the abundance of virulence factors with the organisms carrying these factors. It processes this relationship across all pair comparisons, offering a comprehensive inspection of which taxa may be associated with microbial virulence. Specifically, it aligns contigs to the *Escherichia coli* virulence factors (Ecoli_VF) [63]and virulence factor database (VFDB) [64] using ABRicate [63]. The resulting output tables contain information on hit virulence factors and corresponding contigs. It further joins the taxonomy annotation and abundance of contigs with the hit virulence factors. OUTPOST normalizes counts (equation (6)) before visualizing the distribution of virulence factors among taxa and between groups using cluster heatmaps and histograms separately. *C_v_* is the count of virulence factor *v* in counts set *C*.

|  | $C_{v}^{'}=\left\{ \begin{aligned} \log_{10} C_{v} , &C_{v}>0 \\ \frac{\min\left\{ x\in C \vert x>C_{v} \right\}}{100}, &C_{v}=0 \end{aligned} \right.$ | (6) |
| --- | --- | --- |

**Plasmids analysis**

OUTPOST links the organisms carrying these factors with the abundance of plasmids. It uses ABRicate [63] to align contigs to PlasmidFinder [65]. The analysis and visualization procedures are similar to Virulence factors analysis.

**Antibiotic genes analysis**

OUTPOST associates the abundance of antibiotic genes with the organisms that carry these factors. The contigs are aligned to Resfinder [66], the comprehensive antibiotic resistance database (CARD) [67], ARG-ANNOT [68], MEGARes [69], and AMRFinderPlus database [70] with a similar procedure as Virulence factors analysis.

**Biomarkers analysis**

OUTPOST identifies biomarkers crossing all group pair comparisons and taxonomic levels, with supplementary ANCOM identification (Table S3).

**Log**

OUTPOST logs the execution status, error messages, and output information for each computational step. Completion of a step is marked by a log file with a 'done' suffix, enabling OUTPOST to track progress, automatically skip completed parts and resume from breakpoints. This design also allows users to easily monitor the execution status for debugging. Additionally, it features a benchmark module that records the computational resources and time consumed for each step, facilitating targeted adjustments and development by advanced users.

**OUTPOST report**

Considering the extensive output generated by OUTPOST, an HTML format report is created upon analysis completion to facilitate user comprehension of the results. This report documents all analysis module results with examples, annotating the significance of each analysis module and corresponding file locations. With the aid of the report, users will easily understand the output and identify the information they need for their studies.

**The datasets preparation for software evaluation**

To evaluate the capabilities of OUTPOST, we initiated the assessment by reanalyzing a publicly available cat gut microbiome dataset [20] and comparing it to the original paper. This dataset was a single-batch and two-group experiment, classified into "normal" and "obese" categories. The assembly of the cat microbiome is GCA_022675345.1. We further constructed a more complex and multi-group mammalian gut microbiome dataset. This dataset encompasses 18 samples across six species including pig, dog, cat, horse, buffalo, and camel (Table S2). The dataset size is 295.1Gbp. For reference genomes, we utilized GCF_000181335.3 (cat), GCF_000002285.3 (dog), GCF_000003025.6 (pig), GCA_002863925.1 (horse), GCF_019923935.1 (buffalo), and GCF_000767855.1 (camel). To test the analytical power in therapeutic microbiome projects, OUTPOST was extended to a colorectal neoplasms gut microbiomes dataset retrieved from GMrepo [71]. This dataset includes 32 healthy samples and 30 patients' samples (Table S4) and the size is 450.6Gbp. The reference genome is GCA_000001405.14 (human).

OUTPOST innovatively utilizes meta-analysis to score biomarkers taking advantage of seven information sources. Based on the results of the previous analyses, it sets seven evaluation criteria. These include the ranking of relative abundance, the comparison of inter-group relative abundance, the verification of a sufficiently high LDA score, the ranking of virulence factors' carriage, the ranking of plasmids' carriage, the ranking of antibiotic genes' carriage, and the application of ANCOM (Analysis of Composition of Microbiomes) [72]. ANCOM was designed to detect differentially abundant taxa at the ecosystem level while maintaining robust statistical power. The calculation of the OUTPOST score is detailed in equation (7), where *t* means taxonomy. 1_top relative abundance_ equals to 1 when the conditions indicated by the subscript are met, otherwise equals to 0. We employ scatter plots and volcano plots in this software to visualize the results of ANCOM's analysis.

| $score_{t}=1_{top relative abundance}+1_{statistic significance}+1_{large LDA score}+1_{top virulence factors abundace}+1_{top plasmids factors abundance}+1_{top antibiotic genes abundance}+1_{ANCOM acceptance}$ | (7) |
| --- | --- |

**REFERENCES**

1. Schwengers, Oliver, Andreas Hoek, Moritz Fritzenwanker, Linda Falgenhauer, Torsten Hain, Trinad Chakraborty, Alexander Goesmann. 2020. “ASA3P: An automatic and scalable pipeline for the assembly, annotation and higher-level analysis of closely related bacterial isolates.” *PLoS Comput Biol* 16: e1007134. <https://doi.org/10.1371/journal.pcbi.1007134>

2. Eren, A Murat, Özcan C Esen, Christopher Quince, Joseph H Vineis, Hilary G Morrison, Mitchell L Sogin, Tom O Delmont. 2015. “Anvi'o: an advanced analysis and visualization platform for 'omics data.” *PeerJ* 3: e1319. <https://doi.org/10.7717/peerj.1319>

3. Thomsen, Martin Christen Frolund, Johanne Ahrenfeldt, Jose Luis Bellod Cisneros, Vanessa Jurtz, Mette Voldby Larsen, Henrik Hasman, Frank Moller Aarestrup, Ole Lund. 2016. “A bacterial analysis platform: an integrated system for analysing bacterial whole genome sequencing data for clinical diagnostics and surveillance.” *PLoS One* 11: e0157718. <https://doi.org/10.1371/journal.pone.0157718>

4. Chukamnerd, Arnon, Kongpop Jeenkeawpiam, Sarunyou Chusri, Rattanaruji Pomwised, Kamonnut Singkhamanan, Komwit Surachat. 2023. “BacSeq: A user-friendly automated pipeline for whole-genome sequence analysis of bacterial genomes.” *Microorganisms* 11: 1769. <https://doi.org/10.3390/microorganisms11071769>

5. Narayanasamy, Shaman, Yohan Jarosz, Emilie E L Muller, Anna Heintz-Buschart, Malte Herold, Anne Kaysen, Cedric C Laczny, Nicolas Pinel, Patrick May, Paul Wilmes. 2016. “IMP: a pipeline for reproducible reference-independent integrated metagenomic and metatranscriptomic analyses.” *Genome Biol* 17: 260. <https://doi.org/10.1186/s13059-016-1116-8>

6. Buza, Teresia M, Triza Tonui, Francesca Stomeo, Christian Tiambo, Robab Katani, Megan Schilling, Beatus Lyimo, et al. 2019. “iMAP: an integrated bioinformatics and visualization pipeline for microbiome data analysis.” *BMC Bioinformatics* 20: 374. <https://doi.org/10.1186/s12859-019-2965-4>

7. Pandolfo, Mattia, Andrea Telatin, Gioele Lazzari, Evelien M Adriaenssens, Nicola Vitulo. 2022. “MetaPhage: an Automated Pipeline for Analyzing, Annotating, and Classifying Bacteriophages in Metagenomics Sequencing Data.” *mSystems* 7: e0074122. <https://doi.org/10.1128/msystems.00741-22>

8. Uritskiy, Gherman V, Jocelyne DiRuggiero, James Taylor. 2018. “MetaWRAP-a flexible pipeline for genome-resolved metagenomic data analysis.” *Microbiome* 6: 158. <https://doi.org/10.1186/s40168-018-0541-1>

9. Kultima, Jens Roat, Luis Pedro Coelho, Kristoffer Forslund, Jaime Huerta-Cepas, Simone S Li, Marja Driessen, Anita Yvonne Voigt, Georg Zeller, Shinichi Sunagawa, Peer Bork. 2016. “MOCAT2: a metagenomic assembly, annotation and profiling framework.” *Bioinformatics* 32: 2520-2523. <https://doi.org/10.1093/bioinformatics/btw183>

10. Seemann T, Goncalves da Silva A, Bulach DM, Schultz MB, Kwong JC, Howden BP. 2020. “Nullarbor.” *Github*, <https://github.com/tseemann/nullarbor>

11. Hong, Changjin, Solaiappan Manimaran, Ying Shen, Joseph F Perez-Rogers, Allyson L Byrd, Eduardo Castro-Nallar, Keith A Crandall, William Evan Johnson. 2014. “PathoScope 2.0: a complete computational framework for strain identification in environmental or clinical sequencing samples.” *Microbiome* 2: 33. <https://doi.org/10.1186/2049-2618-2-33>

12. Tamames, Javier, Fernando Puente-Sanchez. 2018. “SqueezeMeta, a highly portable, fully automatic metagenomic analysis pipeline.” *Front Microbiol* 9: 3349. <https://doi.org/10.3389/fmicb.2018.03349>

13. Clarke, Erik L, Louis J Taylor, Chunyu Zhao, Andrew Connell, Jung-Jin Lee, Bryton Fett, Frederic D Bushman, Kyle Bittinger. 2019. “Sunbeam: an extensible pipeline for analyzing metagenomic sequencing experiments.” *Microbiome* 7: 46. <https://doi.org/10.1186/s40168-019-0658-x>

14. Quijada, Narciso M, David Rodriguez-Lazaro, Jose Maria Eiros, Marta Hernandez. 2019. “TORMES: an automated pipeline for whole bacterial genome analysis.” *Bioinformatics* 35: 4207-4212. <https://doi.org/10.1093/bioinformatics/btz220>

15. Beghini, Francesco, Lauren J McIver, Aitor Blanco-Miguez, Leonard Dubois, Francesco Asnicar, Sagun Maharjan, Ana Mailyan, et al. 2021. “Integrating taxonomic, functional, and strain-level profiling of diverse microbial communities with bioBakery 3.” *Elife* 10: e65088. <https://doi.org/10.7554/eLife.65088>

16. Asnicar, Francesco, Andrew Maltez Thomas, Francesco Beghini, Claudia Mengoni, Serena Manara, Paolo Manghi, Qiyun Zhu, et al. 2020. “Precise phylogenetic analysis of microbial isolates and genomes from metagenomes using PhyloPhlAn 3.0.” *Nat Commun* 11: 2500. <https://doi.org/10.1038/s41467-020-16366-7>

17. Truong, Duy Tin, Adrian Tett, Edoardo Pasolli, Curtis Huttenhower, Nicola Segata. 2017. “Microbial strain-level population structure and genetic diversity from metagenomes.” *Genome Res* 27: 626-638. <https://doi.org/10.1101/gr.216242.116>

18. Blanco-Miguez, Aitor, Francesco Beghini, Fabio Cumbo, Lauren J McIver, Kelsey N Thompson, Moreno Zolfo, Paolo Manghi, et al. 2023. “Extending and improving metagenomic taxonomic profiling with uncharacterized species using MetaPhlAn 4.” *Nat Biotechnol* 41: 1633-1644. <https://doi.org/10.1038/s41587-023-01688-w>

19. Franzosa, Eric A, Lauren J McIver, Gholamali Rahnavard, Luke R Thompson, Melanie Schirmer, George Weingart, Karen Schwarzberg Lipson, et al. 2018. “Species-level functional profiling of metagenomes and metatranscriptomes.” *Nat Methods* 15: 962-968. <https://doi.org/10.1038/s41592-018-0176-y>

20. Ma, Xiaolei, Emily Brinker, Emily C Graff, Wenqi Cao, Amanda L Gross, Aime K Johnson, Chao Zhang, Douglas R Martin, Xu Wang. 2022. “Whole-genome shotgun metagenomic sequencing reveals distinct gut microbiome signatures of obese cats.” *Microbiol Spectr* 10: e0083722. <https://doi.org/10.1128/spectrum.00837-22>

21. Sung, Hyuna, Jacques Ferlay, Rebecca L Siegel, Mathieu Laversanne, Isabelle Soerjomataram, Ahmedin Jemal, Freddie Bray. 2021. “Global Cancer Statistics 2020: GLOBOCAN Estimates of Incidence and Mortality Worldwide for 36 Cancers in 185 Countries.” *CA Cancer J Clin* 71: 209-249. <https://doi.org/10.3322/caac.21660>

22. De Almeida, Carolina Vieira, Marcela Rodrigues de Camargo, Edda Russo, Amedeo Amedei. 2019. “Role of diet and gut microbiota on colorectal cancer immunomodulation.” *World J Gastroenterol* 25: 151-162. <https://doi.org/10.3748/wjg.v25.i2.151>

23. Murphy, Neil, Victor Moreno, David J Hughes, Ludmila Vodicka, Pavel Vodicka, Elom K Aglago, Marc J Gunter, Mazda Jenab. 2019. “Lifestyle and dietary environmental factors in colorectal cancer susceptibility.” *Mol Aspects Med* 69: 2-9. <https://doi.org/10.1016/j.mam.2019.06.005>

24. Zheng, Xiaobin, Jinhee Hur, Long H Nguyen, Jie Liu, Mingyang Song, Kana Wu, Stephanie A Smith-Warner, et al. 2021. “Comprehensive Assessment of Diet Quality and Risk of Precursors of Early-Onset Colorectal Cancer.” *J Natl Cancer Inst* 113: 543-552. <https://doi.org/10.1093/jnci/djaa164>

25. Kim, Jaeho, Heung Kyu Lee. 2021. “Potential Role of the Gut Microbiome In Colorectal Cancer Progression.” *Front Immunol* 12: 807648. <https://doi.org/10.3389/fimmu.2021.807648>

26. Arthur, Janelle C, Ernesto Perez-Chanona, Marcus Muhlbauer, Sarah Tomkovich, Joshua M Uronis, Ting-Jia Fan, Barry J Campbell, et al. 2012. “Intestinal inflammation targets cancer-inducing activity of the microbiota.” *Science* 338: 120-123. <https://doi.org/10.1126/science.1224820>

27. Rebersek, Martina. 2021. “Gut microbiome and its role in colorectal cancer.” *BMC Cancer* 21: 1325. <https://doi.org/10.1186/s12885-021-09054-2>

28. Nouri, Roghayeh, Alka Hasani, Kourosh Masnadi Shirazi, Mohammad Reza Alivand, Bita Sepehri, Simin Sotoudeh, Fatemeh Hemmati, Afshin Fattahzadeh, Babak Abdinia, Mohammad Ahangarzadeh Rezaee. 2021. “Mucosa-Associated Escherichia coli in Colorectal Cancer Patients and Control Subjects: Variations in the Prevalence and Attributing Features.” *Can J Infect Dis Med Microbiol* 2021: 2131787. <https://doi.org/10.1155/2021/2131787>

29. Russo, Edda, Giovanni Bacci, Carolina Chiellini, Camilla Fagorzi, Elena Niccolai, Antonio Taddei, Federica Ricci, et al. 2017. “Preliminary Comparison of Oral and Intestinal Human Microbiota in Patients with Colorectal Cancer: A Pilot Study.” *Front Microbiol* 8: 2699. <https://doi.org/10.3389/fmicb.2017.02699>

30. Flemer, Burkhardt, Ryan D Warren, Maurice P Barrett, Katryna Cisek, Anubhav Das, Ian B Jeffery, Eimear Hurley, Micheal O'Riordain, Fergus Shanahan, Paul W O'Toole. 2018. “The oral microbiota in colorectal cancer is distinctive and predictive.” *Gut* 67: 1454-1463. <https://doi.org/10.1136/gutjnl-2017-314814>

31. Wong, Sunny H, Jun Yu. 2019. “Gut microbiota in colorectal cancer: mechanisms of action and clinical applications.” *Nat Rev Gastroenterol Hepatol* 16: 690-704. <https://doi.org/10.1038/s41575-019-0209-8>

32. Tsoi, Ho, Eagle S H Chu, Xiang Zhang, Jianqiu Sheng, Geicho Nakatsu, Siew C Ng, Anthony W H Chan, Francis K L Chan, Joseph J Y Sung, Jun Yu. 2017. “Peptostreptococcus anaerobius Induces Intracellular Cholesterol Biosynthesis in Colon Cells to Induce Proliferation and Causes Dysplasia in Mice.” *Gastroenterology* 152: 1419-1433 e1415. <https://doi.org/10.1053/j.gastro.2017.01.009>

33. Long, Xiaohang, Chi Chun Wong, Li Tong, Eagle S H Chu, Chun Ho Szeto, Minne Y Y Go, Olabisi Oluwabukola Coker, et al. 2019. “Peptostreptococcus anaerobius promotes colorectal carcinogenesis and modulates tumour immunity.” *Nat Microbiol* 4: 2319-2330. <https://doi.org/10.1038/s41564-019-0541-3>

34. Zhao, Liuyang, Xiang Zhang, Yunfei Zhou, Kaili Fu, Harry Cheuk-Hay Lau, Orcid: 0000-0003-3581-2909, Tommy Wai-Yiu Chun, et al. 2022. “Parvimonas micra promotes colorectal tumorigenesis and is associated with prognosis of colorectal cancer patients.” *Oncogene* 41: 4200-4210. <https://doi.org/10.1038/s41388-022-02395-7>

35. Xu, Jun, Min Yang, Dongyan Wang, Shuilong Zhang, Su Yan, Yongliang Zhu, Weichang Chen. 2020. “Alteration of the abundance of Parvimonas micra in the gut along the adenoma-carcinoma sequence.” *Oncol Lett* 20: 106. <https://doi.org/10.3892/ol.2020.11967>

36. Chang, Yuxiao, Ziran Huang, Fengyi Hou, Yuejiao Liu, Likun Wang, Zhen Wang, Yifan Sun, et al. 2023. “Parvimonas micra activates the Ras/ERK/c-Fos pathway by upregulating miR-218-5p to promote colorectal cancer progression.” *J Exp Clin Cancer Res* 42: 13. <https://doi.org/10.1186/s13046-022-02572-2>

37. Wu, Na, Yu-Qing Feng, Na Lyu, Di Wang, Wei-Dong Yu, Yong-Fei Hu. 2022. “Fusobacterium nucleatum promotes colon cancer progression by changing the mucosal microbiota and colon transcriptome in a mouse model.” *World J Gastroenterol* 28: 1981-1995. <https://doi.org/10.3748/wjg.v28.i18.1981>

38. Sun, Chun-Hui, Bin-Bin Li, Bo Wang, Jing Zhao, Xiao-Ying Zhang, Ting-Ting Li, Wen-Bing Li, et al. 2019. “The role of Fusobacterium nucleatum in colorectal cancer: from carcinogenesis to clinical management.” *Chronic Dis Transl Med* 5: 178-187. <https://doi.org/10.1016/j.cdtm.2019.09.001>

39. Abed, Jawad, Naseem Maalouf, Abigail L Manson, Ashlee M Earl, Lishay Parhi, Johanna E M Emgard, Michael Klutstein, et al. 2020. “Colon Cancer-Associated Fusobacterium nucleatum May Originate From the Oral Cavity and Reach Colon Tumors via the Circulatory System.” *Front Cell Infect Microbiol* 10: 400. <https://doi.org/10.3389/fcimb.2020.00400>

40. Fenner, Lukas, Veronique Roux, Pascal Ananian, Didier Raoult. 2007. “Alistipes finegoldii in blood cultures from colon cancer patients.” *Emerg Infect Dis* 13: 1260-1262. <https://doi.org/10.3201/eid1308.060662>

41. Saha, Piu, Beng San Yeoh, Xia Xiao, Rachel M Golonka, Sivarajan Kumarasamy, Matam Vijay-Kumar. 2019. “Enterobactin, an iron chelating bacterial siderophore, arrests cancer cell proliferation.” *Biochem Pharmacol* 168: 71-81. <https://doi.org/10.1016/j.bcp.2019.06.017>

42. Huang, Lingyu, Tongxiang Zou, Wenken Liang, Chune Mo, Jianfen Wei, Yecheng Deng, Minglin Ou. 2023. “High-Throughput Sequencing Reveals That Rotundine Inhibits Colorectal Cancer by Regulating Prognosis-Related Genes.” *J Pers Med* 13: <https://doi.org/10.3390/jpm13030550>

43. Mathlouthi, Nour El Houda, Aicha Kriaa, Leila Ammar Keskes, Moez Rhimi, Radhouane Gdoura. 2022. “Virulence Factors in Colorectal Cancer Metagenomes and Association of Microbial Siderophores with Advanced Stages.” *Microorganisms* 10: <https://doi.org/10.3390/microorganisms10122365>

44. Molder, Felix, Kim Philipp Jablonski, Brice Letcher, Michael B Hall, Christopher H Tomkins-Tinch, Vanessa Sochat, Jan Forster, et al. 2021. “Sustainable data analysis with Snakemake.” *F1000Res* 10: 33. <https://doi.org/10.12688/f1000research.29032.2>

45. Chen, Shifu, Yanqing Zhou, Yaru Chen, Jia Gu. 2018. “fastp: an ultra-fast all-in-one FASTQ preprocessor.” *Bioinformatics* 34: i884-i890. <https://doi.org/10.1093/bioinformatics/bty560>

46. Bolger, Anthony M, Marc Lohse, Bjoern Usadel. 2014. “Trimmomatic: a flexible trimmer for Illumina sequence data.” *Bioinformatics* 30: 2114-2120. <https://doi.org/10.1093/bioinformatics/btu170>

47. Li, Dinghua, Chi-Man Liu, Ruibang Luo, Kunihiko Sadakane, Tak-Wah Lam. 2015. “MEGAHIT: an ultra-fast single-node solution for large and complex metagenomics assembly via succinct de Bruijn graph.” *Bioinformatics* 31: 1674-1676. <https://doi.org/10.1093/bioinformatics/btv033>

48. Nurk, Sergey, Dmitry Meleshko, Anton Korobeynikov, Pavel A Pevzner. 2017. “metaSPAdes: a new versatile metagenomic assembler.” *Genome Res* 27: 824-834. <https://doi.org/10.1101/gr.213959.116>

49. Fu, Limin, Beifang Niu, Zhengwei Zhu, Sitao Wu, Weizhong Li. 2012. “CD-HIT: accelerated for clustering the next-generation sequencing data.” *Bioinformatics* 28: 3150-3152. <https://doi.org/10.1093/bioinformatics/bts565>

50. Gurevich, Alexey, Vladislav Saveliev, Nikolay Vyahhi, Glenn Tesler. 2013. “QUAST: quality assessment tool for genome assemblies.” *Bioinformatics* 29: 1072-1075. <https://doi.org/10.1093/bioinformatics/btt086>

51. Huerta-Cepas, Jaime, Damian Szklarczyk, Davide Heller, Ana Hernandez-Plaza, Sofia K Forslund, Helen Cook, Daniel R Mende, et al. 2019. “eggNOG 5.0: a hierarchical, functionally and phylogenetically annotated orthology resource based on 5090 organisms and 2502 viruses.” *Nucleic Acids Res* 47: D309-D314. <https://doi.org/10.1093/nar/gky1085>

52. Alcock, Brian P, William Huynh, Romeo Chalil, Keaton W Smith, Amogelang R Raphenya, Mateusz A Wlodarski, Arman Edalatmand, et al. 2023. “CARD 2023: expanded curation, support for machine learning, and resistome prediction at the Comprehensive Antibiotic Resistance Database.” *Nucleic Acids Res* 51: D690-D699. <https://doi.org/10.1093/nar/gkac920>

53. Menzel, Peter, Kim Lee Ng, Anders Krogh. 2016. “Fast and sensitive taxonomic classification for metagenomics with Kaiju.” *Nat Commun* 7: 11257. <https://doi.org/10.1038/ncomms11257>

54. Danecek, Petr, James K Bonfield, Jennifer Liddle, John Marshall, Valeriu Ohan, Martin O Pollard, Andrew Whitwham, et al. 2021. “Twelve years of SAMtools and BCFtools.” *Gigascience* 10: <https://doi.org/10.1093/gigascience/giab008>

55. Oksanen, Jari, Gavin L. Simpson, F. Guillaume Blanchet, Roeland Kindt, Pierre Legendre, Peter R. Minchin, R.B. O'Hara, et al. 2024. “vegan: Community Ecology Package.” <https://vegandevs.github.io/vegan/>

56. Asnicar, Francesco, George Weingart, Timothy L Tickle, Curtis Huttenhower, Nicola Segata. 2015. “Compact graphical representation of phylogenetic data and metadata with GraPhlAn.” *PeerJ* 3: e1029. <https://doi.org/10.7717/peerj.1029>

57. Ondov, Brian D, Nicholas H Bergman, Adam M Phillippy. 2011. “Interactive metagenomic visualization in a web browser.” *BMC Bioinformatics* 12: 385. <https://doi.org/10.1186/1471-2105-12-385>

58. Caspi, Ron, Tomer Altman, Richard Billington, Kate Dreher, Hartmut Foerster, Carol A Fulcher, Timothy A Holland, et al. 2014. “The MetaCyc database of metabolic pathways and enzymes and the BioCyc collection of Pathway/Genome Databases.” *Nucleic Acids Res* 42: D459-471. <https://doi.org/10.1093/nar/gkt1103>

59. Kanehisa, Minoru, Yoko Sato, Masayuki Kawashima, Miho Furumichi, Mao Tanabe. 2016. “KEGG as a reference resource for gene and protein annotation.” *Nucleic Acids Res* 44: D457-462. <https://doi.org/10.1093/nar/gkv1070>

60. Mistry, Jaina, Sara Chuguransky, Lowri Williams, Matloob Qureshi, Gustavo A Salazar, Erik L L Sonnhammer, Silvio C E Tosatto, et al. 2021. “Pfam: the protein families database in 2021.” *Nucleic Acids Res* 49: D412-D419. <https://doi.org/10.1093/nar/gkaa913>

61. Bairoch, Amos. 2000. “The ENZYME database in 2000.” *Nucleic Acids Res* 28: 304-305. <https://doi.org/10.1093/nar/28.1.304>

62. Segata, Nicola, Jacques Izard, Levi Waldron, Dirk Gevers, Larisa Miropolsky, Wendy S Garrett, Curtis Huttenhower. 2011. “Metagenomic biomarker discovery and explanation.” *Genome Biol* 12: R60. <https://doi.org/10.1186/gb-2011-12-6-r60>

63. Torsten, Seemann. 2017. “Abricate.” *Github*, <https://github.com/tseemann/abricate>

64. Chen, Lihong, Jian Yang, Jun Yu, Zhijian Yao, Lilian Sun, Yan Shen, Qi Jin. 2005. “VFDB: a reference database for bacterial virulence factors.” *Nucleic Acids Res* 33: D325-328. <https://doi.org/10.1093/nar/gki008>

65. Carattoli, Alessandra, Ea Zankari, Aurora Garcia-Fernandez, Mette Voldby Larsen, Ole Lund, Laura Villa, Frank Moller Aarestrup, Henrik Hasman. 2014. “In silico detection and typing of plasmids using PlasmidFinder and plasmid multilocus sequence typing.” *Antimicrob Agents Chemother* 58: 3895-3903. <https://doi.org/10.1128/AAC.02412-14>

66. Bortolaia, Valeria, Rolf S Kaas, Etienne Ruppe, Marilyn C Roberts, Stefan Schwarz, Vincent Cattoir, Alain Philippon, et al. 2020. “ResFinder 4.0 for predictions of phenotypes from genotypes.” *J Antimicrob Chemother* 75: 3491-3500. <https://doi.org/10.1093/jac/dkaa345>

67. McArthur, Andrew G, Nicholas Waglechner, Fazmin Nizam, Austin Yan, Marisa A Azad, Alison J Baylay, Kirandeep Bhullar, et al. 2013. “The comprehensive antibiotic resistance database.” *Antimicrob Agents Chemother* 57: 3348-3357. <https://doi.org/10.1128/AAC.00419-13>

68. Gupta, Sushim Kumar, Babu Roshan Padmanabhan, Seydina M Diene, Rafael Lopez-Rojas, Marie Kempf, Luce Landraud, Jean-Marc Rolain. 2014. “ARG-ANNOT, a new bioinformatic tool to discover antibiotic resistance genes in bacterial genomes.” *Antimicrob Agents Chemother* 58: 212-220. <https://doi.org/10.1128/AAC.01310-13>

69. Doster, Enrique, Steven M Lakin, Christopher J Dean, Cory Wolfe, Jared G Young, Christina Boucher, Keith E Belk, Noelle R Noyes, Paul S Morley. 2020. “MEGARes 2.0: a database for classification of antimicrobial drug, biocide and metal resistance determinants in metagenomic sequence data.” *Nucleic Acids Res* 48: D561-D569. <https://doi.org/10.1093/nar/gkz1010>

70. Feldgarden, Michael, Vyacheslav Brover, Narjol Gonzalez-Escalona, Jonathan G Frye, Julie Haendiges, Daniel H Haft, Maria Hoffmann, et al. 2021. “AMRFinderPlus and the Reference Gene Catalog facilitate examination of the genomic links among antimicrobial resistance, stress response, and virulence.” *Sci Rep* 11: 12728. <https://doi.org/10.1038/s41598-021-91456-0>

71. Dai, Die, Jiaying Zhu, Chuqing Sun, Min Li, Jinxin Liu, Sicheng Wu, Kang Ning, Li-Jie He, Xing-Ming Zhao, Wei-Hua Chen. 2022. “GMrepo v2: a curated human gut microbiome database with special focus on disease markers and cross-dataset comparison.” *Nucleic Acids Res* 50: D777-D784. <https://doi.org/10.1093/nar/gkab1019>

72. Mandal, Siddhartha, Will Van Treuren, Richard A White, Merete Eggesbo, Rob Knight, Shyamal D Peddada. 2015. “Analysis of composition of microbiomes: a novel method for studying microbial composition.” *Microb Ecol Health Dis* 26: 27663. <https://doi.org/10.3402/mehd.v26.27663>

**Supplementary Figures**

**Figure S1 OUTPOST's validation.** (A) Bar plot of phylum level relative frequency in the microbiota between normal and obsese cat gut microbiome. (B) Cluster heatmap of relative frequency (log normalized) for the top 10 most abundant KEGG Orthology items in the cat gut microbiome. (C) Linear discriminant analysis (LDA) scores of the top featured microbial order in normal (blue) and obese (yellow) cats’ gut microbiotas. (D) Bar plot of species level relative frequency in the mammalian microbiota with multi-group stratification. (E) Example box plots of the relative frequency of Prevotella copri in mammalian microbiota for multi-group comparisons. Statistical significance was calculated using two-tailed Mann-Whitney U test. (F, G) Example computational perturbation experiment using cluster heatmaps of relative frequency (log normalized) for the top 10 most abundant bacterial species. The samples were assigned the correct groups (F) and random pseudo labels (G). The cluster distinction disappeared in pseudo-group results. (H) Box plot of the relative frequency of Escherichia coli, in healthy (blue) and ill (yellow) human microbiotas. Statistical significance was calculated using two-tailed Mann-Whitney U test. (I) Cluster heatmaps of relative frequency (log normalized) for the top 19 most abundant virulence factors in the microbial assembly crossing the species level in therapeutic microbiomes. (J) Bar plots of the top 10 most abundant virulence factors in healthy (blue) and ill (orange) human microbiotas.

**Figure S2 OUTPOST comprehensively characterized the taxonomy atlas.** (A) Bar plot of phylum level relative frequency in the microbiota. (B) Cluster heatmap of relative frequency (log normalized) for the top 20 most abundant bacteria families. (C-H) Box plots of relative frequency for six significant bacteria in normal (blue) and obese (yellow) cat microbiota spanning six taxonomy levels: Firmicutes (phylum), Clostridia (class), Eubacteriales (order), Lachnospiraceae (family), Bifidobacterium (genus), and Lactimicrobium massiliense (species). Statistical significance was calculated using two-tailed Mann-Whitney U test.

**Figure S3 OUTPOST revealed significant different diversities and identified the potential features from the taxonomy and function analysis results.** (A, B) Box plots of alpha diversity in normal (blue) and obese (yellow) cat microbiota at the species level (A) and genus level (B), measured using the Shannon index (A) and Simpson index (B). Statistical significance was calculated using the two-tailed Mann-Whitney U test. (C, D) The PCoA plots of beta diversity between normal (red) and obese (cyan) cat microbiota at species level (C) and genus level (D) using Bray-Curtis distance (C) and Jaccard distance (D). Statistical significance was processed using permutational multivariate analysis of variance (PERMANOVA). (E) Cluster heatmap of relative frequency (log normalized) for the top 20 most abundant KEGG Orthology items in the microbiome. (F) Cluster heatmap of relative frequency (log normalized) for the top 20 most abundant unequal enzymes in the microbiomes. Statistical significance was processed using the two-tailed Mann-Whitney U test. (G, H) Linear discriminant analysis (LDA) scores of the top featured microbial order (G) and genera (H) in normal (blue) and obese (yellow) cats’ gut microbiotas. (I) Linear discriminant analysis (LDA) scores of the top 20 featured enzymes. Taxa with LDA scores greater than 2.0 were included.

**Figure S4 OUTPOST reviewed the virulence factors, antibiotic genes, and plasmids crossing the taxonomy.** (A, B) Cluster heatmaps of relative frequency (log normalized) for the top 10 most abundant virulence factors (A) and antibiotics genes (B) in the microbial assembly crossing the species level (A) and family level (B). gene1 and gene2 are annotated as “Streptomyces cinnamoneus EF-Tu mutants conferring resistance to elfamycin” and “Streptomyces rishiriensis parY mutant conferring resistance to aminocoumarin” in CARD. (C, D) Bar plots of the top 10 most abundant virulence factors (C) and all plasmids (D) in normal (blue) and obese (orange) cats’ microbiotas.

**Figure S5 The multi-group comparison indicated the significantly enriched abundance of Prevotella copri in omnivores, and the ablation experiment enhanced the reliability of the analyzed results.** (A) Bar plot of species level relative frequency in the mammalian microbiota. (B-H) Box plots of the relative frequency of Prevotella copri in omnivore (blue) and herbivore (yellow) microbiota between seven group comparisons. Statistical significance was calculated using two-tailed Mann-Whitney U test. (I, J) Cluster heatmaps of relative frequency (log normalized) for the top 20 most abundant bacterial species. The samples were assigned the correct groups (I) and random incorrect labels (J). (K, L) The PCoA plots of beta diversity between herbivore (red) and omnivore (cyan) microbiota at species level with Jaccard distance. The samples were assigned the correct groups (K) and random incorrect labels (L). Statistical significance was processed using permutational multivariate analysis of variance (PERMANOVA).

**Figure S6 The analysis of therapeutic microbiomes using OUTPOST.** (A−F) Box plots of relative frequency of Escherichia coli (A), Alistipes finegoldii (B), Fusobacterium nucleatum (C), Parvimonas micra (D), Peptostreptococcus anaerobius (E), and Prevotella intermedia (F) in healthy (blue) and ill (yellow) human microbiotas. Statistical significance was calculated using two-tailed Mann-Whitney U test. (G) Cluster heatmaps of relative frequency (log normalized) for the top 19 most abundant virulence factors in the microbial assembly crossing the species level. (H) Bar plots of the top 10 most abundant virulence factors in healthy (blue) and ill (orange) human microbiotas.

**Figure S7 OUTPOST pipeline directed graph.** Each node represents a specific component or task, with directed edges showing the flow of data and sequence of operations, reflecting the overall analytical framework we employed.
